# A pragmatic evaluation of community-based lymphoedema services for individuals at risk of, or living with, cancer-related lymphoedema

**DOI:** 10.1007/s00520-026-10432-4

**Published:** 2026-02-28

**Authors:** Mairéad Cantwell, Fiona Skelly, Patricia Sheehan, Michael O Brien, Bróna Kehoe, Niall Moyna, Andrew McCarren, Dorothy Thomas, Bernie O’Loughlin, Louise Mullen

**Affiliations:** 1 SHE Research Centre, Department of Sport & Health Sciences, The Technological University of the Shannon, Midlands Midwest, University Road, Athlone, Co. Westmeath Ireland; 2 Centre for Health Behaviour Research, South East Technological University, Waterford, Ireland; 3 School of Health and Human Performance, Dublin City University, Dublin, Ireland; 4 School of Computing, Dublin City University, Dublin, Ireland; 5 The National Cancer Control Programme, Dublin, Ireland

**Keywords:** Lymphoedema, Cancer, Community-based care, Evaluation

## Abstract

**Purpose:**

This study examined the long-term effects of standard care lymphoedema treatment, delivered by cancer support centres in community-based settings, on quality of life and self-reported symptom severity among individuals with cancer-related lymphoedema (CRL).

**Methods:**

A single-group pre-post pragmatic study design was adopted. Survivors of cancer referred to community-based cancer support centres were recruited. Participants received a minimum of 3 standard care lymphoedema treatment sessions at the centres with a certified lymphoedema clinician at baseline (T1), 1 month (T2) and 6 months (T3), where core treatment strategies included lymphoedema education to enable self-management, skin care and weight management. Assessments of quality of life and symptom severity were conducted at each visit using limb specific versions of the Lymphoedema Quality of Life (LYMQoL) questionnaire (i.e. arm LYMQoL, leg LYMQoL) as applicable for each person, and a researcher-developed tool of self-reported symptom severity.

**Results:**

One-hundred and twenty survivors of cancer were recruited (mean age (± SD) 59 (± 12y); 90% female). Forty participants (33%) completed the T3 assessment. Linear mixed-model analyses demonstrated significant improvements in arm-CRL QoL (*n* = 78) and leg-CRL QoL (*n* = 18) from T1 to T3 (*p* < 0.05), with small (Cohen’s *d* = 0.24) and large (Cohen’s *d* = 0.85) effect sizes respectively. All 7 self-reported symptom ratings, including pain, swelling and functional restriction, improved from T1–T3.

**Conclusion:**

Standard care lymphoedema treatment, when delivered in community-based cancer support centres, was associated with improvements in QoL and self-reported symptoms among survivors of cancer with arm and leg lymphoedema at 6 months.

**Supplementary Information:**

The online version contains supplementary material available at 10.1007/s00520-026-10432-4.

## Introduction

Lymphoedema, a chronic condition resulting from lymphatic impairment or failure that leads to the accumulation of lymphatic fluid and secondary low-grade inflammation, can occur following cancer treatment as a result of damage to the body’s lymphatic system [1]. Lymphoedema that occurs following oncologic intervention (i.e. cancer-related lymphoedema (CRL)) is classified as secondary lymphoedema and can arise weeks, months and years following cancer treatment [2, 3].

Prevalence estimates of CRL have been hindered by the absence of a universally accepted definition and associated diagnostic criteria [4]. Letellier and colleagues [4] have reported differences in CRL incidence across cancer types, including rates of 2–74% for survivors of breast cancer and rates of 71–90% for survivors of head and neck cancer. In Ireland, prevalence estimates ranging from 10 to 75% have been reported, depending on the site of cancer occurrence [5].

CRL is associated with significant burden for survivors of cancer which may continue long after diagnosis and include reduced psychosocial well-being, social confidence and quality of life (QoL), increased rates of depression, anxiety and stress, difficulties engaging in activities of daily life, including the ability to work, and financial challenges as a result of productivity losses and increased out-of-pocket costs [6, 7]. Due to increases in cancer incidence and survival rates, CRL incidence is likely to also grow in the future and healthcare systems need to prepare to meet the needs of the expanding community of survivors of cancer who have CRL [4].

The gold standard for CRL management is complete decongestive therapy (CDT) which has multiple goals, including educating individuals about their condition, reducing limb volume and fibrosis in the affected limb, improving functional capacity, optimising QoL and supporting effective self-management by the patient, including the adoption and/or maintenance of healthy lifestyle habits (e.g. regular physical activity participation) [1, 2, 5]. However, access to such treatment by individuals with CRL can be challenging and may be influenced by a number of factors including delayed diagnosis, disparities among healthcare professionals’ knowledge of CRL, criteria for diagnosis and resources and services to support its management, inequity in service provision across geographical regions, as well as extended wait times to access services [8, 9]. The financial cost of treatment for CRL is also significant and can be influenced by differences in government subsidies, healthcare insurance policy cover and the magnitude of direct individual out-of-pocket costs [8]. There are also practical challenges regarding the delivery of CDT within community-based settings, including increased service demand and complexity in patient profiles where longer treatment periods are needed to achieve results [10].

In Ireland, lymphoedema service provision has previously been described as insufficient and inequitable, where lack of funding has been cited as a major challenge to the continued provision of such services [11]. Patients have described the lack of available services to meet the needs of individuals living with lymphoedema, where existing services were under-resourced and oversubscribed [11].

To address the gaps in the provision of services for the management of CRL, a national public provider of cancer services provided funding to cancer support centres (CSCs) to provide lymphoedema treatment to individuals with CRL. The aim of this investigation was to conduct a pragmatic evaluation of this service, which was delivered by CSCs in community-based settings, to determine its impact on QoL (primary outcome) and self-reported lymphoedema-symptom severity (secondary outcome), among individuals living with CRL.

## Methods

### Study design

This single-group, pre-post pragmatic study evaluated the standard care (SC) lymphoedema service that was provided to patients at community-based CSCs who received grant aid from the National Cancer Control Programme (NCCP) (*n* = 15). Recruitment, which occurred via convenience sampling on a rolling basis, commenced in May 2022. Data collection was completed by April 2024.

### Setting and participants

Staff at CSCs recruited participants to take part in the study. Where possible, details of the study were shared with participants (i) when contact was made in person, by telephone or email to schedule a baseline assessment for lymphoedema treatment or (ii) at patient in-take. The staff explained the study to patients verbally and provided a plain language statement to interested individuals. Patients were asked to read this information in advance of their baseline assessment, during which informed consent procedures were completed if the patient agreed to participate.

The inclusion criteria for the study were: (i) adults (≥ 18 years old) who were at risk of, or living with CRL, and (ii) had been referred, or self-referred, to a participating community-based CSC for lymphoedema treatment. Individuals at risk of lymphoedema were included in order to capture the impact of treatment in the context of prevention/early-stage intervention. Individuals with an unstable medical condition for whom lymphoedema treatment was contraindicated were excluded (e.g. patients with acute cellulitis). Ethical approval for the study was granted by the Athlone Institute of Technology Research Ethics Committee (REC 20220304).

### Lymphoedema treatment

Participants’ treatment was aligned with SC for the prevention and/or management of lymphoedema as described by The Health Service Executive (HSE) [5] and was dependent upon the individualised treatment plan advised by the lymphoedema clinician following completion of a baseline assessment (Supplementary File 1). Core prevention measures within SC included lymphoedema awareness education, strength training and physical activity (PA), weight management and skin care. In addition, compression and elevation may have been included to improve venous and lymph drainage as part of the management of lymphoedema in the context of SC. Further details regarding the lymphoedema treatment provided as part of SC are described in Supplementary File 2.

Lymphoedema treatment was provided by certified lymphoedema clinicians, who in this context refers to all lymphoedema therapists, including nurses and health and social care professionals. All therapists were certified with a recognised lymphoedema training programme (e.g. Vodder, Leduc, Casley Smith, Klose).

Participants completed a baseline assessment (referred to as T1) and follow-up assessments at 1 and 6 months referred to as T2 and T3, respectively. Assessments were also conducted after a period of intensive treatment (T2b) for patients that required it. However, the number of sessions completed was dependent upon the severity of the patient’s lymphoedema and their individualised treatment plan. All assessments were guided by standardised assessment/re-assessment forms, in line with the HSE All-Ireland Lymphoedema Guidelines [5] (See Supplementary Files 1 and 3). Where participants could not attend for a face-to-face assessment at T2 or T3, questionnaires, as described below, were administered over the phone or posted/emailed to participants. Participants that completed 3 assessments (T1, T2, T3) are described as Group 1, and participants that completed 4 assessments (T1, T2, T2b, T3) are described as Group 2. Trial completion was defined based on the number of Group 1 participants who completed the T3 assessment.

### Outcome measures

Participant demographic information (e.g. age, gender, cancer diagnosis) was collected as part of the baseline assessment. The International Society of Lymphology (ISL) staging system [12] was used to assess lymphoedema severity on a scale where ‘0’ denoted latent/subclinical lymphoedema and ‘III’ denoted lymphostatic elephantiasis. Information pertaining to the individual’s height, weight, body mass index, history of oedema, current symptoms, past medical history, problem list and goals of treatment were also collected.

#### Primary outcome variable

The primary outcome variable was QoL, as measured by the Lymphoedema Quality of Life (LYMQoL) questionnaire. In order to reinforce the clinical relevance of findings, this questionnaire was purposefully chosen given that it is a condition-specific measure of QoL. It is a valid and reliable QoL assessment tool which can be used for lymphoedema of the limbs both in clinical assessment and as an outcome measure [13]. It is a patient-completed questionnaire which includes questions that cover 4 domains, namely symptoms, body image/appearance, function and mood. Two versions of the questionnaire are available for both arm (21 items) and leg (22 items) lymphoedema. A total score for each domain is calculated by adding all scores together and dividing by the total number of questions answered [14]. If fewer than 50% of the items were answered, the whole domain is scored as 0. The ‘overall QoL’ item is scored 0–10, where 0 indicates poor QoL and 10 indicates excellent QoL.

#### Secondary outcome variable

Symptom severity was assessed using a researcher-developed tool which asked participants to self-report the perceived severity on a 0–10 point scale of 7 lymphoedema-related symptoms, where 0 indicates no presence of the symptom and 10 indicates extreme severity. The 7 symptoms were: functional restriction, heaviness, reduced range of movement (ROM), pain, skin changes, swelling and paraesthesia. Further detail regarding the development of this tool is described in Supplementary File 4.

### Data analysis

This was a pragmatic evaluation of an existing service and so no formal sample size calculation was applied. Rather data collection ran for a 2-year period and an analysis of the data collected in that time took place.

CSC staff (i.e. centre managers, administrative staff, lymphoedema clinicians) were responsible for data collection and entry. Within the below results section, the figures used to calculate percentages for different variables are specified where necessary to account for missing data.

Statistical analyses were conducted in IBM SPSS Statistics (version 28.0). Staging of lymphoedema was treated as an ordinal variable and a Friedman test was used to investigate changes between T1, T2 and T3. Numerical responses were analysed using SPSS where descriptive statistics, including means and standard deviations, were reported. Symptom-ratings were aggregated for all participants and were not analysed in the context of arm- or leg-lymphoedema specific differences. A linear mixed-model analysis was used to investigate longitudinal changes between T1, T2 and T3. This test is an ideal approach when several participants are studied across a series of time points, as the model does not exclude participants who have missing data and does not need complete data sets in order to be used [15, 16]. Additionally, the model is suitable for studies in which data collection has not been conducted at equally spaced timepoints [15]. The Akaike Information Criterion and Bayesian Information Criterion were used as metrics to identify the best-fit model for each outcome measure. The model was analysed for autoregressive, compound symmetry, diagonal, toeplitz, toeplitz heterogeneous and unstructured variance structures. To optimise model fit, the variance structure was selected individually for each outcome measure, as detailed in Supplementary File 5. Time was treated as a repeated measures and a fixed effect in the model. To investigate the impact of the additional visit which group 2 received (i.e. T2b), group was included as a fixed effect in the model. The main effect for time and group and the interaction effect of time*group were investigated. Bonferroni-adjusted post hoc stratified analysis comparing estimated marginal means at each timepoint were performed for all outcome measures that indicated a significant main effect for time. Cohen’s *d* was calculated. A value of 0.1 to 0.49 was defined as a small effect size, 0.5 to 0.79 as medium, and 0.8 to 1.0 as large [17]. Effect sizes and their corresponding confidence intervals were also examined to support interpretation of the practical significance of results. A simple effects analysis was conducted to explore any significant group or time*group effects.

## Results

### Participants

Participant baseline characteristics are presented in Table 1. Participants’ (*n* = 118) mean age was 59 ± 12 years (range 19–85 years), 90% (*n* = 109) were female and breast cancer was the most reported cancer diagnosis (80%; *n* = 91). 6%, 4% and 2% of participants had had gynaecological, melanoma or neck cancer, respectively. Fifty-one per cent (*n* = 43) of participants reported a history of oedema. Referrals for lymphoedema treatment at the CSC were primarily from consultants (23%; *n* = 27) and general practitioners (21%; *n* = 25).

**Table 1:** Participant demographics

Variable	*n* (%)
Gender (*n* = 120)	
* Male*	8 (7)
* Female*	109 (90)
* Did not say*	3 (3)
Cancer diagnoses (*n* = 114)	
* Breast*	91 (80)
* Gynaecological*	8 (6)
* Melanoma*	5 (4)
* Neck*	2 (2)
* Prostate*	1 (1)
* Sarcoma*	1 (1)
* Bowel*	1 (1)
* Kidney*	1 (1)
* Endometrial*	1 (1)
* Leukemia*	1 (1)
* Thyroid*	1 (1)
* Other*	1 (1)
Referral source (*n* = 119)	
* Consultant*	27 (23)
* GP*	25 (21)
* Nurse*	14 (12)
* Physio*	9 (8)
* Other*	44 (37)
History of Oedema (*n* = 85)	
* Yes*	43 (51)
* No*	42 (49)

Categorical variables are presented as *n* (%).

One hundred and twenty participants were recruited to the study between May 2022 and December 2023. All participants consented to participate. Sixty-four and fifteen participants completed assessments at T2 and T2b, respectively. Trial completion was defined based on the number of Group 1 participants who completed the T3 assessment, which was 33% (*n* = 40). The average number of days between the T1 and T2 assessment was 43 days (± 27; range = 13–151 days). The average number of days between T2 and T3 assessment was 160 days (± 49; range = 55–265 days). It was anticipated that T3 assessments would take place approximately 24–26 weeks after T1 assessments. The average number of weeks between the T1 and T3 assessment was 29 weeks (± 5; range = 22–44 weeks).

### Lymphoedema care: problems, goals and treatment plan

At T1, 21%, 39% and 38% of participants had stage 0, 1 or 2 lymphoedema, according to the International Society of Lymphology (ISL) Staging System, respectively. Additionally, 1 participant had Stage 3, and 1 participant had Stage 4, lymphoedema at baseline. Friedmans test did not identify any significant differences in lymphoedema severity according to the ISL Staging System [11] between T1, T2, and T3 (χ^2^ (3) = 0.94, df = 2, *p* = 0.815).

Table 2 summarises (i) the lymphoedema-related problems that participants reported experiencing at baseline, (ii) the treatment goals set by the lymphoedema clinician, and (iii) the treatment modalities that were used as part of participants’ individualised treatment plan. The most prevalent lymphoedema-related problems reported by participants at baseline were increased limb volume (46%), poor knowledge of lymphoedema and its management (43%) and altered limb shape (32%). Treatment goals mirrored effective strategies to address the problems identified where improvements in knowledge, limb volume and arm range of movement (AROM) were targeted for 35%, 33% and 21% of participants, respectively. Exercise (55%), skin care (45%) and teaching participants how to do lymph drainage (42%) were the most frequently used treatment strategies.

**Table 2:** Lymphoedema-related problems, goals and treatment strategies identified and targeted at T1 (*n* = 120)

Problems	% (*n*)	Goals	% (*n*)	Treatment plan	% (*n*)
Increased limb volume	46 (55)	Increase knowledge	35 (42)	Exercise	55 (66)
Poor knowledge	43 (51)	Reduce limb volume	33 (40)	Skin care	45 (54)
Altered limb shape	32 (39)	Improve AROM	21 (25)	Teach lymph drainage	42 (50)
Tissue fibrosis	16 (19)	Restore limb shape	18 (21)	Education	35 (42)
Decreased strength	15 (18)	Able to carry out SLD	17 (20)	Compression garments	33 (40)
Pain	14 (17)	Independent exercise	15 (18)	Manual lymphatic drainage	18 (22)
Decreased range of motion	13 (15)	Pain reduction	15 (18)	Other (e.g. Deep oscillation, Kinesio taping, wraps, physio touch)	14 (17)
Reduced exercise	11 (13)	Improve strength	15 (18)	Multi-layer lymph bandaging	10 (12)
Poor Skin	8 (10)	Improve skin integrity	14 (17)	Teach self-bandaging wrapping	4 (5)
		Maintain stable lymphoedema	13 (16)		
		Carry out skin regime	13 (15)		
		Tissue softening	10 (12)		
		Patient able to don doff	8 (9)		
		Able to carry out MLLB	6 (7)		

Abbreviations: *AROM*, Active Range of Motion; *SLD*, Simple Lymphatic Drainage; *MLLB*, Multi-Layer Lymphoedema Bandaging.

### Primary outcome variable: LYMQoL Questionnaire

The linear mixed models for primary and secondary outcomes were run including T1, T2 and T3. The main effect for time and group and the interaction effect of time*group was investigated to determine the impact of T2b, which is also reported below.

Table 3 presents the mixed-model test results for arm and leg LYMQoL questionnaires at T1, T2 and T3.

**Table 3:** Linear mixed-model analyses of lymphoedema variables at T1, T2 and T3

Outcome variables	n	T1	T2	T3	Df	F	sig	Cohens *d*
*Arm LYMQoL*								
* Overall QoL*	78	6.3 ± 0.2	6.1 ± 0.3	7.1 ± 0.2	(2, 43)	7.4	**0.002**	0.24
* Symptoms*	77	2.2 ± 0.1	1.9 ± 0.1	1.8 ± 0.1	(2, 43)	6.4	**0.004**	0.27
* Function*	75	1.5 ± 0.1	1.4 ± 0.1	1.3 ± 0.1	(2, 39)	1.8	0.176	
* Appearance*	78	2.0 ± 0.1	1.8 ± 0.1	1.7 ± 0.1	(2, 94)	2.2	0.119	
* Mood*	75	1.9 ± 0.1	2.0 ± 0.1	1.8 ± 0.1	(2, 71)	1.4	0.242	
*Leg LYMQoL*								
* Overall QoL*	18	3.9 ± 1.1	4.7 ± 1.2	6.3 ± 0.6	(2, 5)	10.5	**0.018**	0.85
* Function*	14	1.9 ± 0.3	1.8 ± 0.3	2.0 ± 0.3	(2, 3)	6.2	0.084	
* Appearance*	17	2.9 ± 0.5	2.5 ± 0.5	2.6 ± 0.6	(2, 4)	0.3	0.759	
* Symptoms*	18	2.5 ± 0.5	2.0 ± 0.5	1.9 ± 0.5	(2, 7)	1.2	0.353	
* Mood*	18	1.8 ± 0.5	1.6 ± 0.5	1.3 ± 0.5	(2, 8)	2.9	0.113	
*Self-Reported Symptoms*								
* Heaviness*	118	4.3 ± 0.4	3.9 ± 0.6	2.5 ± 0.5	(2, 71)	4.9	**0.010**	0.61
* Reduced ROM*	118	2.7 ± 0.4	2.5 ± 0.4	1.3 ± 0.4	(2, 170)	30.2	** < 0.001**	0.53
* Tingling, Pins & Needles*	118	2.4 ± 0.4	1.4 ± 0.5	0.9 ± 0.3	(2, 53)	7.4	**0.001**	0.41
* Pain*	117	2.8 ± 0.4	1.3 ± 0.6	1.1 ± 0.5	(2, 78)	6.1	**0.003**	0.62
* Functional Restriction*	118	2.7 ± 0.4	2.4 ± 0.4	1.7 ± 0.5	(2, 170)	20.3	** < 0.001**	0.24
* Skin Changes*	117	1.8 ± 0.4	1.0 ± 0.4	0.6 ± 0.7	(2, 151)	11.5	** < 0.001**	0.30
* Swelling*	118	4.7 ± 0.4	3.6 ± 0.5	3.3 ± 0.5	(2, 68)	5.4	**0.007**	0.50

Abbreviations:* LYMQoL*, Lymphoedema Quality of Life questionnaire; *QoL*, Quality of Life; *ROM*, Range of Motion.

Data at each timepoint presented as estimated marginal means ± standard error. Numbers in **BOLD** indicate *p* ≤ 0.05.

#### Upper limb—Arm LYMQoL

**Fig. 1:**
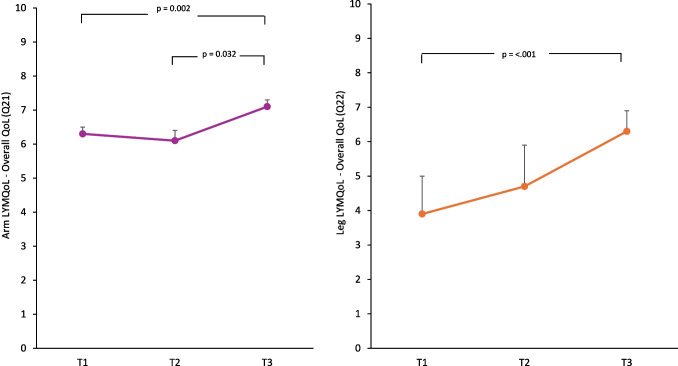
Arm LYMQoL—Overall QoL—and Leg LYMQoL—Overall QoL (T1 – T3) pairwise comparison. Data presented as estimated marginal means and standard error

Overall QoL (Q21) (*n* = 78) improved significantly between T1 and T3 (*p* = 0.002) and T2 to T3 (*p* = 0.032), with a small effect size (Cohen’s *d* = 0.24) (Fig. 1). Symptoms (*n* = 77) reduced significantly between T1 and T3 (*p* = 0.003) with a small effect size (Cohen’s *d* = 0.27). No statistically significant differences were observed for function (*n* = 75), appearance (*n* = 78) or mood (*n* = 75). There were no significant group or group*time effects found for any domain of the arm LYMQoL.

#### Lower limb—Leg LYMQoL

Overall QoL (Q22) (*n* = 18) improved significantly between T1 and T3 (*p* < 0.001) with a large effect size (Cohen’s *d* = 0.85) (Fig. 1). No statistically significant difference was observed for function (*n* = 14), appearance (*n* = 17), symptoms (*n* = 18) or mood (*n* = 18). There were no significant group or group*time effects found for any domain of the leg LYMQoL.

### Secondary outcome variable: self-reported symptom ratings

Table 3 presents the mixed-model test results for self-reported symptoms at T1, T2 and T3.

**Fig. 2:**
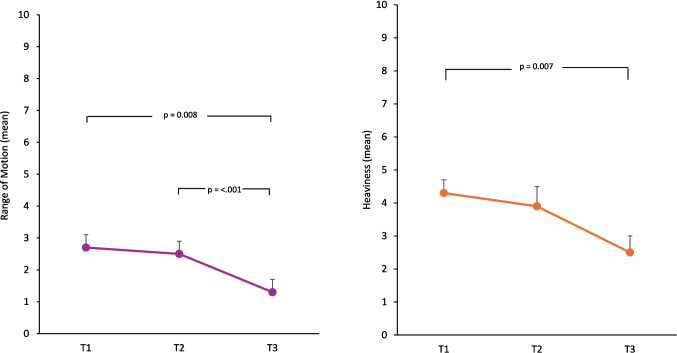
Self-reported lymphoedema symptom ratings: range of motion and heaviness (T1–T3) pairwise comparison. Data presented as estimated marginal means and standard error

There was a significant improvement between T1 and T3 for ratings for heaviness (*p* = 0.007) and ROM (*p* = 0.008), with a medium effect size (Fig. 2). ROM ratings also significantly improved between T2 and T3 (*p* < 0.001) with a medium effect size. Swelling and pain ratings also significantly decreased between T1 and T2 (*p* = 0.042; *p* = 0.029) and T1 and T3 (*p* = 0.019; *p* = 0.011), with a medium effect size. Tingling, pins and needles (*p* = 0.002) and skin changes (*p* = 0.003) ratings significantly improved between T1 and T3 with a small effect size. Skin changes ratings also significantly decreased between T2 and T3 (*p* = 0.020), with a small effect size. Participants’ self-reported functional restriction ratings significantly improved between T1 and T3 (*p* = 0.027) and T2 and T3 (*p* < 0.001), with a small effect size.

There was a significant main effect for group on skin changes (*p* = 0.034), indicating an overall mean difference in skin change scores when averaged across the 3 timepoints. Simple effects analysis did not indicate differences between groups for any specific timepoints. Although statistically significant, the between-group difference for skin changes was modest, with a mean difference of 1.2 points (SE = 0.5) on the 0–10 scale.

**Fig. 3:**
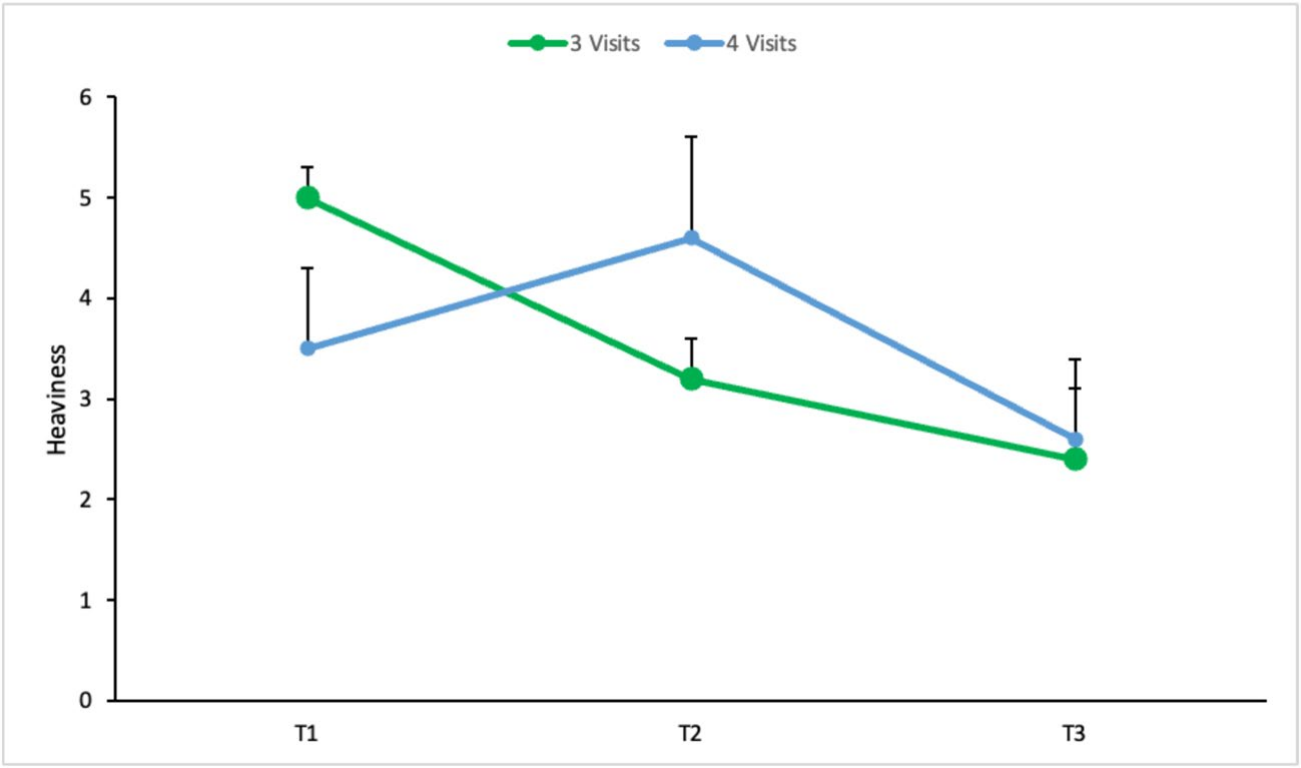
Heaviness (T1–T3) for Group 1 (3 visits) and Group 2 (4 visits). Data presented as estimated marginal means and standard error

A significant interaction effect for time*group was found for heaviness (*p* = 0.022) indicating the change in heaviness over time differed between the two groups. A simple effects analysis was conducted to identify if group difference existed at any specific timepoint. Although the overall interaction was significant, none of the individual timepoint comparisons reached statistical significance. Group 2 had an observed worsening in heaviness at T2 (Fig. 3).

## Discussion

This study is novel as it examined the impact of SC for the management of CRL on the QoL and self-reported symptom severity of survivors of cancer as part of a pragmatic evaluation of an existing service delivered within a real-world community setting. This research makes an important contribution to the scientific literature in this area given the limited evidence base which has examined the effectiveness of lymphoedema treatment delivered in real-world community-based practice. The results showed that SC lymphoedema treatment was associated with improvements in arm and leg CRL overall QoL and self-reported symptoms, including pain, swelling and ROM, among survivors of cancer at 6 months. Symptoms reduced significantly, as measured by the arm LYMQoL questionnaire, and this finding is consistent with the improvements reported in participants’ self-reported symptom severity ratings.

CDT is considered standard of care in the treatment of CRL given its effectiveness in improving the condition, including symptom management, limb volume and disability burden [5, 18]. The findings from this research are consistent with this assertion of CDT being effective for the treatment of CRL given the improvements in QoL and self-reported symptom severity observed. Much of the evidence used to support the recommendations for the use of CDT in the treatment of CRL within the clinical guidelines referenced was derived from multiple randomised clinical trials or meta-analysis. The findings from this research provide important evidence to bolster this recommendation given that this study was conducted in a real-world setting and thus has high external validity.

It is important to also recognise that while statistically significant, the improvements in overall QoL, as measured by the arm LYMQoL questionnaire, were small. Statistically significant improvements in function, appearance or mood did not occur. However, it should be noted that mean scores for these domains were classified across the ‘not at all’ and ‘a little’ ratings, which suggested that clients’ lymphoedema did not have an extensive negative impact on these constructs, and the margin for improvement may have been narrower as a result. Despite this, factors which may have contributed to the lack of improvement observed across these domains are presented below.

Firstly, lymphoedema self-management regimen adherence rates of < 25% to > 75% have been reported previously [19, 20]. Self-management plans for lymphoedema can encompass multiple modalities of care depending on the severity of lymphoedema, which can lead to significant burden for the individual [19, 21]. This can be further exacerbated by the regularity with which self-management strategies need to be performed and the fact that they need to be maintained for the remainder of life [21, 23]. Many individuals also experience psychosocial and/or psychological distress during and after cancer, and such factors have been correlated with low adherence to self-care treatment plans for CRL [21–24]. Higher adherence to the treatment plan prescribed may have been required to achieve improvements in function, mood and appearance.

Secondly, treatment visits did not always occur at the intended times of 1 and 6 months and this may have attenuated the observed improvements in QoL and its subdomains.

Finally, given that improvements in depression, function and general health status have been reported previously among women with breast CRL who received one hour of CDT, 5 days per week for 4 weeks [25], a greater volume of lymphoedema treatment, beyond the 3–4 treatment visits that occurred within this investigation, may also have been required to improve mood, function and appearance, and achieve a greater magnitude of improvement in QoL.

The sample size was not sufficiently large enough to determine the impact of the T2b visit on QoL and/or self-reported symptom ratings. This small sample size at T2b was anticipated given that participants were likely to be offered this visit in cases of more severe lymphoedema which were less common. Treatment was associated with improvements in arm and leg overall QoL and self-reported symptoms at 6 months which indicates that this cohort benefitted from the treatment. Consequently, the T2b visit should still be offered by clinicians as part of the service to individuals deemed to require it.

### Strengths and limitations

The pragmatic research design and sophisticated statistical analysis conducted are both strengths of this investigation. The former enabled valuable evidence regarding the effectiveness of an existing lymphoedema service to be collected, and provides important insights to support service optimisation, particularly given the inclusion of multiple centres. Efforts to optimise the service in light of the findings presented can get underway with immediate effect and thus increases the study’s external validity. The latter enabled the sample size for the analysis to be maximised and thus provides greater confidence in the findings reported.

It was not possible to include a control group given that this study was a pragmatic evaluation of an existing service and thus withholding care would have raised ethical concerns. However, the absence of a control group for comparison is a limitation of the study, as is the small sample size of individuals with leg CRL and the high rate of attrition observed. Participating CSCs were responsible for data collection and entry, which was a new process for the majority of centres who had little-to-no prior experience of engaging in impact monitoring. This contributed to a higher volume of missing data for some variables (e.g. body mass index) which inhibited reporting in some instances. Participants’ adherence to their self-management treatment plan, further detailed information regarding treatment exposure (i.e. the no. of participants who received full CDT vs. initial treatment and maintenance only) and rates of non-uptake and drop-out, as well as factors influencing the variability in the timing of assessments were not reported on and also represent limitations within this study. A ceiling effect in terms of upper limits for improvement may also have occurred in the context of mood, appearance and function among individuals with arm CRL, given the lower ratings which spanned ‘not at all’ and ‘a little’ at all assessment points.

### Recommendations for future research

Future research should include measurement of objective (e.g. circumferential limb measurements) and psychosocial (e.g. depression) outcomes to determine the impact of SC lymphoedema treatment, delivered within community-based settings for individuals with CRL, beyond QoL and symptom severity to provide a more comprehensive assessment of treatment effectiveness. Participant adherence to their self-management plan, and lymphoedema clinician inter-rater reliability, should also be assessed and reported. Validity and reliability of the symptom-rating tool should be determined. A wait-list control group should be considered where practicable to strengthen the study design and fully elucidate the impact of SC, however the challenge of adopting such a study design in a community-based service setting is also recognised. CSCs should be supported to ensure that data quality is optimised and missing data is reduced through the adoption of strategies including standardised data-entry training, centralised monitoring and periodic audits.

## Conclusion

SC lymphoedema treatment delivered in community-based CSCs was associated with improved QoL and lymphoedema-related symptoms, for individuals living with arm and leg CRL, after 6 months. Findings provide important evidence to advocate for continued service delivery, and should inform future policy development and equitable resource allocation to support the increasing number of individuals living with the burden of CRL.

## Supplementary Information

Below is the link to the electronic supplementary material.

Supplementary Material (PDF 0.97 MB)

## Data Availability

The data that support the findings of this study are available from the authors and project funders, The National Cancer Control Programme, but restrictions apply to the availability of these data, which were used under license for the current study, and so are not publicly available. Data are however available from the authors upon reasonable request and with permission of The National Cancer Control Programme.

## Abbreviations

*AROM*: Active Range of Motion
*CDT*: Complete Decongestive Therapy
*CRL*: Cancer-related Lymphoedema
*CSC*: Cancer Support Centre
*HSE*: Health Service Executive
*ISL*: The International Society of Lymphology
*LYMQoL*: Lymphoedema Quality of Life Questionnaire
*MLLB*: Multi-Layer Lymphoedema Bandaging
*NCCP*: National Cancer Control Programme
*QoL*: Quality of Life
*ROM*: Range of Motion
*SC*: Standard Care
*SLD*: Simple Lymphatic Drainage
