# Supplementary material for: A pragmatic evaluation of community-based lymphoedema services for individuals at risk of, or living with, cancer-related lymphoedema
Source: Support Care Cancer. 2026 Feb 28;34(3):259. doi: 10.1007/s00520-026-10432-4 (PMC12950083; doi:10.1007/s00520-026-10432-4)
Supplement: Supplementary file 1 — Supplementary Material (PDF 0.97 MB) [file 520_2026_10432_MOESM1_ESM.pdf]

Supplementary File 1: Baseline Lymphoedema Assessment Form & Treatment Plan

Date of Initial Assessment: DOB: AGE:

NAME:
ADDRESS: PHONE:

MOBILE:

GP: GP phone:

Consultant Name, Location and Speciality

Medical Card Number if applicable:

Referral Source: ☐ GP ☐ Consultant ☐ Nurse ☐ Physio ☐ Other
Name and details: ___________________________

Are you participating in any research projects (outside of this project) or receiving any therapeutic intervention which could influence your lymphoedema, quality of life and/or lymphoedema-related symptoms?
Yes / No (lymphoedema clinician to circle)

If yes, please provide details below.

____
____
____

STAFF SAFETY ALERTS:

History of Oedema (Include: location,progression, cellulitis, previous management of oedema/cellulitis, aggravating/relieving factors)	Consent for assessment / ECR ◻
	Date of lymphoedema onset:
Patient's Perception:<br>Family History of Oedema Yes / No____<br>Ethnicity:	

<u>Current Symptoms</u>

Please indicate whether each symptom, as self-reported by the patient, is ✓ = present or x = absent. If symptoms are present, please circle the number on the 10 point scale which best reflects the patient's symptom status. An example has been included at the start of the table below.

<u>Example:</u>

✓	**Functional Restriction**

No Functional Restriction	0	1	2	3	4	5 (circled)	6	7	8	9	10	Severe Functional Restriction

	**Functional Restriction**

No Functional Restriction	0	1	2	3	4	5	6	7	8	9	10	Severe Functional Restriction

	**Skin changes (tight / shiny)**

No Skin Changes	0	1	2	3	4	5	6	7	8	9	10	Extreme Skin Changes

	**Heaviness**

No Heaviness	0	1	2	3	4	5	6	7	8	9	10	Extreme Heaviness

	**Swelling (difficulty with clothing / rings)**

No Swelling	0	1	2	3	4	5	6	7	8	9	10	Extreme Swelling

	**Reduced ROM**

ROM is Normal	0	1	2	3	4	5	6	7	8	9	10	ROM is severely reduced

Tingling, pins and needles, paraesthesia

No tingling, pins and needs, paraesthesia	0	1	2	3	4	5	6	7	8	9	10	Extreme tingling, pins and needs, paraesthesia

**Pain (related to lymphoedema)**	Site: ________________________<br>Description: _________________

No Pain	0	1	2	3	4	5	6	7	8	9	10	Worst Pain Imaginable

Cancer Related Lymphoedema

Cancer diagnosis:		
Date(s) of surgery:		
Regional lymph node clearance □ (Level 1 / 2 / 3)	Sentinel node biopsy □	Nodes +ve/removed (eg 2/20):

Post operative complications:		Seroma	Details:
		Cording	
		Infection	
		Delayed Wound Healing	

Hormonal Therapy (regime, date started)
Chemotherapy (regime, no. of cycles, date completed)
Radiotherapy (site, date completed, length of treatment)

Past Medical History (√ = *present; x= absent add additional conditions*)

	**Allergies** eg penicillin, latex, elastoplast	

		Varicose Veins	
Sleep Apnoea		Renal Failure	
Heart Failure		DVT	
Phlebitis		PE	
Hypertension		Cholecystectomy	
Thyroid		Skin Disorder	
PVD		Rheumatoid arthritis	
Diabetes		Osteo arthritis	
Obesity		Neuro disorder	

Further Details of PMH:

General Precautions ***(Contraindications)***

	Heart failure	□**uncontrolled**	□ controlled	
	Deep vein thrombosis	□ **acute**	□ chronic	
	Phlebitis/cellulitis	□ **acute**	□ history of	No.of episodes in past year: Prophylaxis: Y/N Hospital admissions
	Renal failure Stage 3+	□ **acute**	□ chronic	Stage:

Neck MLD Precautions ***(Contraindications)***

	Thyroid	□ hypo	□ ***hyper***	
	Cardiac arrhythmia			
	Hypersensitivity of carotid sinus			

Deep abdominal MLD Precautions ***(Contraindications)***

	Abdominal aortic aneurysm	

	Abdominal pain (unexplained)	
	Abdominal radiotherapy	
	Abdominal surgery (recent)	
	Diverticulitis/Bowel disease	
	Pregnancy/Menses	

MLLB Precautions ***(Contraindications)***				
	Peripheral arterial disease	□ ***ABPI <0.5***	□ ABPI 0.6-0.8	□ ABPI >1.3

**Medication (**especially medications linked to oedema**)**

**Investigations** (CT/Lymphoscintography/MRI/ICG/duplex scan)

Social History

Occupation	Smoker Y / N
Hobbies/ Interests/ Relevant social	
Accommodation (including type, access, stairs, bathroom/toilet etc)	
Sleeps in: □ bed □ chair	
Services / Carer Support	
Functional limitations	

Height (m)	Weight (Kg)	BMI (kg/m^2)

ROM

**Upper Limb**	**ROM**	**Lower Limb**	**ROM**
Neck		L/Spine	
Shoulder		Hip	
Elbow		Knee	
Wrist/Hand		Ankle/Foot	

Swelling *(below charts to be completed as appropriate (i.e. upper limb measurements for arm lymphoedema and lower limb for leg lymphoedema)*

CIRCUMFERENTIAL UPPER LIMB MEASUREMENTS				(Affix identification label here)<br>URN:<br>Family name:<br>Given name(s):<br>Address:<br>Date of birth: Sex: ☐ M ☐ F	
Position of patient:	☐ Sitting	Variations:	☐ Abd. 90	Variations:	
Board levels:	☐ Right	Tip 3rd finger:	Mid point MCP:	Mid point MCP radial:	Mid ulna styloid:
	☐ Left	Tip 3rd finger:	Mid point MCP:	Mid point MCP radial:	Mid ulna styloid:
Dominance:	☐ Right ☐ Left ☐ Ambidextrous				

DATE										
Weight/ Height/ BMI										
Side										
MCP										
Mid palm										
Ulna styloid										
10cm										
20cm										
30cm										
40cm										
50cm										
60cm										
Sum										
Difference										
Fingers 1.										
2.										
3.										
4.										
5.										
Therapist and time of day										

CIRCUMFERENTIAL LOWER LIMB MEASUREMENTS				(Affix identification label here)<br>URN:<br>Family name:<br>Given name(s):<br>Address:<br>Date of birth: Sex: ☐ M ☐ F
Position of patient:	☐ Supine		☐ Variations:	
Board levels:	Right	MTP med: lat:	TMT med: lat:	Mid-point malleoli med: lat:
	Left	MTP med: lat:	TMT med: lat:	Mid-point malleoli med: lat:
Dominance:	☐ Right ☐ Left ☐ Ambidextrous			

DATE										
Weight/height/BMI										
Side										
MTP										
TMT										
DAC										
10cm										
20cm										
30cm										
40cm										
50cm										
60cm										
70cm										
80cm										
Sum										
Difference										
Toes 1.										
2.										
3.										
4.										
5.										

Key	√/x	**Observations** (√= *present; x = absent; include key for body chart as necessary*:	
		Broken skin (site)	
		Cancerous skin changes	
		Cellulitis	
		Discolouration	
		Dry	
		Fatty	
		Fibrotic	
		Fragile/Taut/Shiny	
		Fungal infections	
		Hyperkeratosis	
		Misshapen Limb	
		Lymphangiectasia	(lymph blisters)
		Lymphorrhea	
		Non-pitting oedema	
		Papillomatosis	
		Pitting oedema	
		Scarring	
		Skin folds	
		Sensation	Intact / altered
		Stemmers sign	RIGHT:Neg/ positive LEFT: Negative/ positive
		Temperature	RIGHT: Normal/cold/warm LEFT: Normal/cold/warm
		Genital oedema □ Y □ N □ N/A	

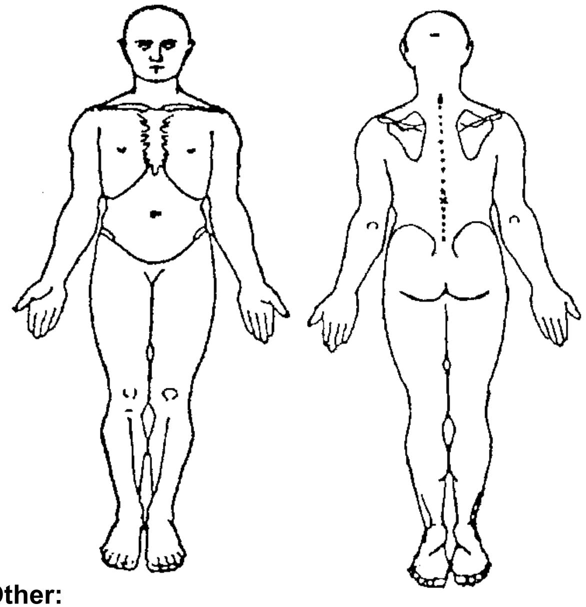

Other:

Are there any signs of vascular disease? □ Yes □ No
Doppler required □ Yes □ No

Diagnosis (If there is more than 1 cause, please rank in order (1=most significant cause)

Type of cancer		Breast
		Gynaecological
		Melanoma
		Prostate
		Head and neck
		Other
Malignant Disease		Infiltrative carcinoma
		Lymph node metastases
		Lymphoma
		Pressure from large tumours
Trauma and tissue damage		Large/circumferential wounds
		Radiotherapy
		Scarring
		Lymph node excision

ISL Lymphoedema Staging			
	0 - Latent/subclinical lymphoedema where impaired lymph transport and subtle changes are present, but swelling is not evident		II - Limb elevation alone rarely reduces swelling; pitting is manifest but may not be apparent later in Stage II due to the development of subcutaneous fat and fibrosis
	I - Accumulation of protein rich fluid that subsides with limb elevation and may cause pitting		III - Lymphostatic elephantiasis – pitting absent due to progressive development of fat and fibrosis, trophic skin changes and warty overgrowths develop

Problem List and Goals of Treatment - discussed and agreed with patient Y / N

	**Problem List**		**Goals of Treatment**		
	Poor knowledge of lymphoedema		Increase knowledge of lymphoedema		Pain reduction
	Increased limb volume		Reduce limb volume		Improve AROM
	Altered limb shape		Restore normal limb shape		Improve strength
	Tissue fibrosis		Improve skin integrity		Able to carry out SLD
	Poor skin condition		Patient able to carry out skincare regime		Able to carry-out MLLB
	Reduced activity/exercise		Independent with exercise		
	Pain		Patient able to don/doff		
	Decreased range of movement		Maintain stable lymphoedema		
	Decreased strength		Tissue softening		

Treatment Plan:

**Maintenance**		**CDT**		Other	
Exercise		Multi-layer Lymphoedema Bandaging<br>Frequency: Duration:		Physiotouch	
Skin care		Bandaging cautions leaflet		Intermittent Pneumatic Compression	
Compression garments		Manual Lymphatic Drainage<br>Frequency: Duration:		Deep Oscillation	
Teach simple lymphatic drainage		Wraps		Kinesiotaping	
Teach self-bandaging/wrapping					
Education					

Recommended onward referral through GP for:

	Activity Resources		Palliative Care		Physiotherapy		Sleep Clinic
	Dermatology		Obesity Clinic		Social Work		
	Dietetics		Occupational Therapy		Podiatry		Vascular
	Genetics		Oncology		Psychology		Wound Care/Tissue Viability

Compression Garment Information - Type, Class

Discharge summary sent to Referrer - Yes / No

Clinician Name:
Signature: **Date:**

Supplementary File 2: *Lymphoedema Treatment Overview*

Participants' treatment regime was aligned with current usual/standard care for the prevention and/or management of lymphoedema and was dependent upon the individualised treatment plan advised by the lymphoedema clinician. Core prevention measures within standard care included 1) strength training and physical activity (PA), 2) weight management (e.g. clinicians should consider referral to a dietitian or local weight management service if clinically indicated), 3) skin care and 4) lymphoedema awareness education. The goals of lymphoedema management focused on improving venous and lymph drainage. Compression, elevation, PA and skincare may have been included as part of the management of lymphoedema in the context of standard care. Compression may have included bandaging and/or garments.

Management of lymphoedema was split into two phases:

Intensive Phase (Phase 1)

In this phase of management, the short-term aims were to:

- Restore maximal functional independence and postural imbalance
- Provide psychological support
- Reduce risk of infection
- Reduce the volume of swelling until it becomes stable
- Improve limb shape where possible
- Improve skin condition
- Meet patient's subjective goals
- Educate patients in understanding their condition and rationale for treatment
- Collaborative working with other services if wounds/lymphorrhoea are present

Treatment in this phase may have included:

(1) Lymphoedema education to enable self-management
(2) Strength training and PA
(3) Skin care
(4) Weight management
(5) Compression garments/wraps (if volumetric difference 5-10%)
(6) Compression bandaging
(7) Manual lymphatic drainage / simple lymphatic drainage
(8) Intermittent pneumatic compression (IPC),
(9) Mobilising tissue, fascia release technique
(10) Kinesio taping
(11) Deep ossilation therapy
(12) Negative pressure therapy

Maintenance Phase (Phase 2)

In this phase of management, the long-term aims were to:

- Maintain reduction in swelling
- Maintain improvement in limb shape
- Maintain skin integrity

Maintenance phase components may have included:

(1) Lymphoedema education to further develop self-management skills
(2) Simple lymphatic drainage (SLD)
(3) Self-bandaging (potentially also nocturnal)
(4) Compression garments (potentially also nocturnal)
(5) IPC,
(6) Strength training and PA
(7) Weight management
(8) Monitoring and self-application skin care
(9) Referral to local and/or external resources for lifestyle/behaviour support (e.g. diet, activity).

Family/carers may have been required to support both the intensive and maintenance phases of care. Occasionally some people may have required a short period of further intensive therapy. This may have been necessary after a medical event, such as, after a deep vein thrombosis, wound or an infection when they were unable to wear compression. As stated in the current lymphoedema clinical guidelines (i.e. Health Service Executive: All-Ireland Lymphoedema Guidelines 2022 for the Diagnosis, Assessment and Management of Lymphoedema), the treatment provided should always be determined by the clinical presentation and clinical judgement. CDT may be considered:

- in patients with a relative volumetric difference of greater than 10% between the affected limb/segment and the unaffected limb/segment, taking into consideration hand dominance and its impact on volume differences.
- in patients with bilateral limb swelling, based on clinical judgment and patient-reported symptoms.

One to one review sessions with the therapist may have been required periodically to maintain the patient/carer's competency to self multi-layer lymphoedema bandaging and SLD and review compression options. The number of visits patients received as part of their treatment may have varied depending on their individualised treatment plan. Following the initial assessment, the follow-up assessment form was completed by the therapist at each subsequent visit (Supplementary File 3). Please see below for details of contraindications to compression therapy and manual lymphatic drainage (MLD).

A comprehensive assessment and on-going review is essential to minimise patient risk. All lymphoedema clinicians worked within their competency and followed current lymphoedema clinical guidelines (i.e. Health Service Executive: All-Ireland Lymphoedema Guidelines 2022 for the Diagnosis, Assessment and Management of Lymphoedema).

Compression therapy (compression garments and/or bandages) is integral to the successful management of lymphoedema. All therapists were competent in measuring and prescribing garments and vascular

assessment. All patients, where relevant, were given written instructions to prevent any adverse effects (see below).

Compression Bandaging Precautions:

Participants were advised to remove the bandages and contact their therapist if they experienced any of the following:

- Increased shortness of breath that was not normal for them
- Significant increased swelling above the bandages
- Extreme itch/skin irritation
- Fever/flu like symptoms
- Increased pain (especially in the toes and fingers) that was not normal for them
- Fingers/toes turned blue or purple
- Un-resolving numbness or new tingling sensations
- Bandages slipped down significantly

Useful hints:

- Patients were encouraged to moisturise their skin at night after removing the garment as cream can make it difficult to put on.
- Put the garment on first thing in the morning before swelling has a chance to accumulate, but not straight after a shower/bath as dampness may make putting the garment on difficult.
- Patients should also have ensured there were no creases and that the garment was evenly distributed - wearing rubber gloves can help gripping and to smooth it out.
- If there was any discomfort or chaffing, patients were advised to talk to their lymphoedema clinician if it didn't settle.
- Always exercise with the garment on.
- Lymphoedema clinicians advised patients on how long to wear their garment but usually it was worn all day, where it could have been taken off at night when patients were lying down/resting.
- Patients should have had 2 garments - one to wash and one to wear. Read the washing instruction before discarding packaging. Most can be machine washed but should not go in the drier. Non-biological washing products should be used and fabric conditioner avoided. Try and alternate to maintain longevity and elasticity.
- Garments should not be folded over, cut or altered in anyway. Replace damaged garments as soon as possible.

Patients should REMOVE the garment if:

- Their skin turns blue/purple,
- They experience prolonged numbness or pins and needles,
- They feel short of breath
- It makes their swelling worse
- It fits incorrectly

- If it causes pain or skin irritation
- If they have cellulitis

Lymphoedema can fluctuate despite best attempts to maintain stability. If patients noticed a change in their lymphoedema they should not have hesitated to contact their lymphoedema clinician.

Contraindications for compression therapy

- Patients with severe PAD with any of the following: ABPI <0.5; toe pressure <30 mmHg; transcutaneous oxygen pressure < 20 mmHg
- Suspected compression of an existing epifascial arterial bypass
- Severe or uncontrolled cardiac insufficiency
- Confirmed allergy to compression material
- Severe diabetic neuropathy with sensory loss or microangiopathy with the risk of skin necrosis (this may not apply to inelastic compression exerting low levels of sustained compression pressure, or modified compression).

Contraindications to manual lymphatic drainage (MLD)

MLD should be avoided in acute cellulitis, lymphangitis and erysipelas, unstable heart failure and untreated Deep Venous Thrombosis (DVTs).

There are precautions that should be considered after full assessment and risk screening which include malignancy, pregnancy, hypotension, unstable hypertension and renal failure.

Supplementary File 3: Patient Follow-Up Assessment Form

Patient Name: DOB: Date:

Consent to review ☐

Subjective

Changes to HPC and medical history

Cellulitis episodes since last appointment (including management)

Yes ☐ (requires review) details...

No ☐ (no action)

Skin Care

Complete skin care daily Yes ☐ No ☐

Emollient used..
....

Garments

Are garments still being worn? Yes ☐ (no action) No ☐ details……………………………………

Are garments comfortable? Yes ☐ (no action) No ☐ (requires review)

Any areas of irritation? Yes ☐ (requires review) No ☐ (no action)

Is swelling well controlled? Yes ☐ (no action) No ☐ (requires review)

How often are garments worn? Every day ☐ Most days ☐ Occasionally ☐ Never ☐

Are garments worn at all times except overnight? Yes ☐ No ☐

If No, how many hours per day are garments worn?…………………………………………………………………

Any difficulties with applying or removing hosiery Yes ☐ (requires review) No ☐ (no action)

Exercise

Daily exercise complete...

Weight change

Significant gain or loss Yes ☐ details.. (requires review)

No ☐ (no action)

Patient Compliance

Has the patient been compliant with the treatment plan advised (please tick)? Yes ☐ No ☐

If no, what challenges has the patient experienced?

____

____

____

Treatment

Assessment

Plan

Current Symptoms

Please indicate whether each symptom, as self-reported by the patient, is ✓ = present or x = absent. If symptoms are present, please circle the number on the 10 point scale which best reflects the patient's symptom status. An example has been included at the start of the table below.

Example:

✓	**Functional Restriction**

No Functional Restriction	0	1	2	3	4	5 (circled)	6	7	8	9	10	Severe Functional Restriction

	**Functional Restriction**

No Functional Restriction	0	1	2	3	4	5	6	7	8	9	10	Severe Functional Restriction

	**Skin changes (tight / shiny)**

No Skin Changes	0	1	2	3	4	5	6	7	8	9	10	Extreme Skin Changes

	**Heaviness**

No Heaviness	0	1	2	3	4	5	6	7	8	9	10	Extreme Heaviness

Swelling (difficulty with clothing / rings)

No Swelling	0	1	2	3	4	5	6	7	8	9	10	Extreme Swelling

Reduced ROM

ROM is Normal	0	1	2	3	4	5	6	7	8	9	10	ROM is severely reduced

Tingling, pins and needles, paraesthesia

No tingling, pins and needs, paraesthesia	0	1	2	3	4	5	6	7	8	9	10	Extreme tingling, pins and needs, paraesthesia

Pain (related to lymphoedema)

Site: ____________________

Description: ______________

No Pain	0	1	2	3	4	5	6	7	8	9	10	Worst Pain Imaginable

ISL Lymphoedema Staging			
	0 - Latent/subclinical lymphoedema where impaired lymph transport and subtle changes are present, but swelling is not evident		II - Limb elevation alone rarely reduces swelling; pitting is manifest but may not be apparent later in Stage II due to the development of subcutaneous fat and fibrosis
	I - Accumulation of protein rich fluid that subsides with limb elevation and may cause pitting		III - Lymphostatic elephantiasis – pitting absent due to progressive development of fat and fibrosis, trophic skin changes and warty overgrowths develop

Swelling *(below charts to be completed as appropriate (i.e. upper limb measurements for arm lymphoedema and lower limb for leg lymphoedema)*

CIRCUMFERENTIAL UPPER LIMB MEASUREMENTS

(Affix identification label here)
URN:
Family name:
Given name(s):
Address:
Date of birth: Sex: ☐ M ☐ F

**Position of patient:**	☐ Sitting	Variations:	☐ Abd. 90	Variations:	
**Board levels:**	☐ Right	Tip 3rd finger:	Mid point MCP:	Mid point MCP radial:	Mid ulna styloid:
	☐ Left	Tip 3rd finger:	Mid point MCP:	Mid point MCP radial:	Mid ulna styloid:
**Dominance:**	☐ Right ☐ Left ☐ Ambidextrous				

DATE										
Weight/ Height/ BMI										
**Side**										
MCP										
Mid palm										
Ulna styloid										
10cm										
20cm										
30cm										
40cm										
50cm										
60cm										
**Sum**										
Difference										
Fingers 1.										
2.										
3.										
4.										
5.										
Therapist and time of day										

CIRCUMFERENTIAL LOWER LIMB MEASUREMENTS

(Affix identification label here)
URN:
Family name:
Given name(s):
Address:
Date of birth: Sex: ☐ M ☐ F

**Position of patient:**	☐ Supine		☐ Variations:				
**Board levels:**	Right	MTP med:	lat:	TMT med:	lat:	Mid-point malleoli med:	lat:
	Left	MTP med:	lat:	TMT med:	lat:	Mid-point malleoli med:	lat:
**Dominance:**	☐ Right ☐ Left ☐ Ambidextrous						

**DATE**										
Weight/height/BMI										
**Side**										
MTP										
TMT										
DAC										
10cm										
20cm										
30cm										
40cm										
50cm										
60cm										
70cm										
80cm										
**Sum**										
**Difference**										
**Toes 1.**										
**2.**										
**3.**										
**4.**										
**5.**										

Signature____ **Date** ________________

Supplementary File 4: Development of the Symptom-Rating Tool

The symptom-rating tool was developed to capture meaningful data pertaining to changes in lymphoedema-related symptoms over time as part of this study. The VAS scale for pain was being used as part of routine care prior to study commencement. The VAS has been demonstrated as a valid and reliable scale and an appropriate measure of pain in clinical practice (Begum and Hossain, 2019). Lymphoedema clinicians also screened for the presence or absence of 6 other symptoms, namely functional restriction, heaviness, reduced range of movement (ROM), skin changes, swelling and paraesthesia. In partnership with staff from the cancer support centres, and with guidance from the National Lymphoedema Service, the team agreed to apply the VAS scale to the 6 symptoms named above and to use the data from this symptom rating tool as the secondary outcome measure for the study. This approach ensured more meaningful data regarding the changes in symptom severity over time, beyond their presence or absence, could be obtained. This approach was also in-keeping with the pragmatic nature of the trial and ensured alignment with current practice given that these symptoms were already assessed as part of routine care.

Reference: Begum, M.R., Hossain, M.A. Validity and reliability of visual analogue scale (VAS) for pain measurement. J Med Case Rep Rev. 2019; 2(11): 394-402.

Supplementary File 5

Mixed-Model Analysis: Variance Structure Selected for Each Outcome Measure

Outcome variables	Variance Structure
Arm LYMQoL	
Overall QoL	Unstructured
Symptoms	Toeplitz: Heterogeneous
Function	Toeplitz: Heterogeneous
Appearance	Diagonal
Mood	Compound Symmetry
Leg LYMQoL	
Overall QoL	Toeplitz: Heterogeneous
Function	Autoregressive
Appearance	Compound Symmetry
Symptoms	Compound Symmetry
Mood	Toeplitz: Heterogeneous
Self-Reported Symptoms	
Heaviness	Autoregressive
Reduced ROM	Toeplitz
Tingling, Pins & Needles	Toeplitz: Heterogeneous
Pain	Autoregressive
Functional Restriction	Toeplitz
Skin Changes	Unstructured
Swelling	Autoregressive

LYMQoL = Lymphoedema Quality of Life questionnaire; QoL = Quality of Life; ROM = Range of Motion
